# Risk of Adverse Neonatal Outcomes After Combined Prenatal Cannabis and Nicotine Exposure

**DOI:** 10.1001/jamanetworkopen.2024.10151

**Published:** 2024-05-07

**Authors:** B. Adam Crosland, Bharti Garg, Gretchen E. Bandoli, Ava D. Mandelbaum, Sarena Hayer, Kimberly S. Ryan, Lyndsey E. Shorey-Kendrick, Cindy T. McEvoy, Eliot R. Spindel, Aaron B. Caughey, Jamie O. Lo

**Affiliations:** 1 Division of Maternal Fetal Medicine, Department of Obstetrics and Gynecology, Oregon Health & Science University, Portland; 2 Department of Obstetrics and Gynecology, Oregon Health & Science University, Portland; 3 Department of Pediatrics, University of California, San Diego; 4 Division of Neuroscience, Oregon National Primate Research Center, Beaverton; 5 Division of Neonatology, Department of Pediatrics, Papé Family Pediatric Research Institute, Oregon Health & Science University, Portland

## Abstract

**Importance:**

The prevalence of cannabis use in pregnancy is rising and is associated with adverse perinatal outcomes. In parallel, combined prenatal use of cannabis and nicotine is also increasing, but little is known about the combined impact of both substances on pregnancy and offspring outcomes compared with each substance alone.

**Objective:**

To assess the perinatal outcomes associated with combined cannabis and nicotine exposure compared with each substance alone during pregnancy.

**Design, Setting, and Participants:**

This retrospective population-based cohort study included linked hospital discharge data (obtained from the California Department of Health Care Access and Information) and vital statistics (obtained from the California Department of Public Health) from January 1, 2012, through December 31, 2019. Pregnant individuals with singleton gestations and gestational ages of 23 to 42 weeks were included. Data were analyzed from October 14, 2023, to March 4, 2024.

**Exposures:**

Cannabis-related diagnosis and prenatal nicotine product use were captured using codes from *International Classification of Diseases, Ninth Revision, Clinical Modification*, and *International Statistical Classification of Diseases, Tenth Revision, Clinical Modification*.

**Main Outcome and Measures:**

The main outcomes were infant and neonatal death, infants small for gestational age, and preterm delivery. Results were analyzed by multivariable Poisson regression models.

**Results:**

A total of 3 129 259 pregnant individuals were included (mean [SD] maternal age 29.3 [6.0] years), of whom 23 007 (0.7%) had a cannabis-related diagnosis, 56 811 (1.8%) had a nicotine-use diagnosis, and 10 312 (0.3%) had both in pregnancy. Compared with nonusers, those with cannabis or nicotine use diagnoses alone had increased rates of infant (0.7% for both) and neonatal (0.3% for both) death, small for gestational age (14.3% and 13.7%, respectively), and preterm delivery (<37 weeks) (12.2% and 12.0%, respectively). Moreover, risks in those with both cannabis and nicotine use were higher for infant death (1.2%; adjusted risk ratio [ARR], 2.18 [95% CI, 1.82-2.62]), neonatal death (0.6%; ARR, 1.76 [95% CI, 1.36-2.28]), small for gestational age (18.0%; ARR, 1.94 [95% CI, 1.86-2.02]), and preterm delivery (17.5%; ARR, 1.83 [95% CI, 1.75-1.91]).

**Conclusions and Relevance:**

These findings suggest that co-occurring maternal use of cannabis and nicotine products in pregnancy is associated with an increased risk of infant and neonatal death and maternal and neonatal morbidity compared with use of either substance alone. Given the increasing prevalence of combined cannabis and nicotine use in pregnancy, these findings can help guide health care practitioners with preconception and prenatal counseling, especially regarding the benefits of cessation.

## Key Points

**Question:**

Is in utero exposure to cannabis and nicotine in combination associated with greater adverse outcomes than exposure to either substance alone during pregnancy?

**Findings:**

In this population-based cohort study of more than 3.1 million pregnant individuals, combined use of cannabis and nicotine products in pregnancy was associated with an increased risk of maternal and neonatal morbidity compared with use of either substance alone, including infant and neonatal death, infants small for gestational age, and preterm delivery.

**Meaning:**

These findings suggest that more effective public health measures and counseling prior to conception and during pregnancy are warranted to mitigate the potential for adverse offspring outcomes from combined prenatal cannabis and nicotine use.

## Teaser

This cohort study assesses the perinatal outcomes associated with combined prenatal cannabis and nicotine product use compared with the use of either substance alone among pregnant individuals in the US.

## Introduction

With growing legalization, prenatal cannabis use has become increasingly prevalent in the US and can be as high as 30% in younger, urban populations.^1,2^ There is concern for adverse pregnancy outcomes, given that Δ9-tetrahydrocannabinol (THC, the main psychoactive component of cannabis) can readily cross the placenta.^3^ The available evidence suggests that prenatal cannabis exposure is associated with stillbirth, neonatal intensive care unit (NICU) admissions, impaired fetal neurodevelopment, preterm delivery, and infants small for gestational age.^4,5,6^

Of particular concern, half of individuals who use cannabis in pregnancy also use tobacco or nicotine products,^7^ and combined use of cannabis and nicotine products in pregnancy has increased over time.^8^ Numerous studies^8,9,10,11,12,13^ have demonstrated the detrimental effects of prenatal tobacco exposure, primarily nicotine exposure, on perinatal outcomes, including prematurity, low birth weight, and stillbirth. Currently there is a paucity of epidemiological data regarding the effects of combined prenatal exposure to both cannabis and nicotine products. Emerging evidence indicates that cannabis use exacerbates the negative impacts of nicotine product exposure, and dual abstinence may result in better outcomes following cessation.^14,15^ The limited existing literature suggests that combined use of cannabis and nicotine products is associated with adverse maternal health outcomes, including an increased risk of cannabis use disorder and worsened mental health.^14,15,16,17,18,19,20^ In addition, prenatal exposure to cannabis and nicotine products has been associated with decreased birth weight^21^ and altered infant neurobehavior compared with unexposed infants.^22^

While studies have individually examined the consequences of cannabis and nicotine exposure, their combined impact on maternal and neonatal outcomes remains largely unknown. The dearth of information regarding combined use of cannabis and nicotine in pregnancy is particularly problematic given that combined use will likely continue to increase as more states legalize recreational cannabis, and the limited existing evidence demonstrates reason for concern. A comprehensive understanding of the interplay between these substances is essential for guiding health care practitioners in providing informed counseling to optimize antenatal care. Therefore, this study assesses the perinatal outcomes associated with prenatal combined use of cannabis and nicotine products compared with the use of either substance alone.

## Methods

### Cohort Selection

This retrospective, population-based cohort study used linked vital statistics and hospital discharge data in California (2012 to 2019). Linkage of vital statistics and hospital discharge was performed at Oregon Health & Science University. We linked vital statistics (birth and fetal death certificates) maintained by the California Department of Public Health and patient discharge data maintained by Health Care Access and Information. Probabilistic linkage was used to link the 2 datasets; it used a combination of available common data elements and variables that were present in both datasets, such as the neonate’s birth date, the neonate’s sex, birth hospital, the patients’ residential zip code, and hospital county. Of the 3 643 173 birth certificate records, 94% of the vital statistics data were linked to both maternal and neonatal hospital discharge records. A comparison of the patients in the analytic sample and those who were excluded because of nonlinkage is provided in eTable 1 in Supplement 1. The final dataset included all maternal and neonatal characteristics derived from birth and death certificates, as well as diagnosis and procedure codes recorded using *International Classification of Diseases, Ninth Revision, Clinical Modification* (*ICD-9-CM*) from January 1, 2012, to September 30, 2015, and *International Statistical Classification of Diseases, Tenth Revision, Clinical Modification* (*ICD-10-CM*) from October 1, 2015, to December 31, 2019, from hospital discharge records. This study was approved by the Oregon Health & Science University Institutional Review Board and the Committee for the Protection of Human Subjects of the Health and Human Services Agency in the State of California, which waived the need for informed consent owing to the use of deidentified registry data. The study followed the Strengthening the Reporting of Observational Studies in Epidemiology (STROBE) reporting guideline.

### Inclusion and Exclusion Criteria

We included pregnant individuals with singleton gestation and gestational ages of 23 to 42 weeks. Participants who self-reported American Indian or Alaska Native, Asian or Native Hawaiian or Other Pacific Islander, Black (non-Hispanic), Hispanic, and White (non-Hispanic) race and ethnicity were included. Exclusion criteria were multiple births and gestational ages less than 23 weeks and greater than 42 weeks. We also excluded patients who self-reported more than 1 race or race other than the aforementioned categories because this group is heterogenous.

### Primary Exposure

Our primary exposures were cannabis and/or nicotine product use during pregnancy, which were identified using birth certificates and *ICD-9-CM* and *ICD-10-CM* codes. Cannabis use was identified using *ICD-9-CM* (304.3 and 305.2) and *ICD-10-CM* (F12) codes; nicotine use was captured using birth certificate data and *ICD-9-CM* (305.1 and 649.0) and *ICD-10-CM* (O99.33 and F17) codes. Pregnant individuals were classified into 4 categories: control (no cannabis or nicotine use), cannabis use, nicotine use, and combined cannabis and nicotine use.

### Demographic Characteristics and Comorbidities

Maternal characteristics were retrieved from the birth certificate or hospital discharge data and included maternal age, parity, race and ethnicity, educational attainment, insurance, and prepregnancy body mass index (BMI; calculated as weight in kilograms divided by height in meters squared). Maternal age was grouped into 3 categories of younger than 20, 20 to 34, or 35 years or older. Parity was defined as nulliparous or multiparous. Self-reported maternal race and ethnicity were classified according to the aforementioned inclusion and exclusion criteria; these data are potentially more affected by target resources and counseling. Maternal educational attainment was stratified as attendance through high school or less or having attended some college. Insurance status was grouped as private and public, self-pay, or uninsured. Prepregnancy BMI was tabulated as underweight (<18.5), normal weight (18.5-24.9), overweight (25.0-29.9), or obesity (≥30.0). The number of prenatal visits was also examined categorically as less than 5 and 5 or more based on previous literature.^23^ We used *ICD-9-CM* and *ICD-10-CM* codes to determine alcohol-related use disorder and mental health (anxiety, depression, or bipolar) disorders. These codes are provided in eTable 2 in Supplement 1. Maternal comorbidities included chronic hypertension and preexisting diabetes (eTable 2 in Supplement 1).

### Outcomes

Adverse perinatal outcomes were defined using *ICD-9-CM* or *ICD-10-CM* codes or birth certificate information. Maternal outcomes of interest included hypertensive disease (eg, gestational hypertension and preeclampsia with or without severe features), and preterm (gestational age <37 weeks) and very preterm (gestational age <32 weeks) delivery. Severe maternal morbidity (SMM) was defined using a published and previously validated list of *ICD* codes from the Centers for Disease Control and Prevention.^24,25^ Patients were classified as having SMM based on the presence of at least 1 of the following indicators: acute myocardial infarction, aneurysm, acute kidney failure, acute respiratory distress syndrome, amniotic fluid embolism, cardiac arrest or ventricular fibrillation, conversion of cardiac rhythm, disseminated intravascular coagulation, eclampsia, heart failure, puerperal cerebrovascular disorders, pulmonary edema or acute heart failure, severe anesthesia complications, sepsis, shock, sickle cell disease with crisis, air and thrombotic embolism, transfusion with blood products, hysterectomy, temporary tracheostomy, and ventilation. Because blood transfusion is a major marker of SMM and occurs much more commonly than other SMM markers,^25^ we conducted analyses for 2 versions of the SMM variable: one with and one without blood transfusion.

Neonatal outcomes of interest included infant death, neonatal death, postneonatal death, NICU admission, respiratory distress syndrome, hypoglycemia, small for gestational age, and bronchopulmonary dysplasia. The source of these outcomes is described in eTable 2 in Supplement 1. Small for gestational age was defined using a published algorithm and defined as a birth weight less than the 10th percentile for gestational age.^26^ Infant deaths were defined as deaths within 1 year of birth; neonatal deaths, deaths within 28 days after birth; and postneonatal deaths, deaths between 28 and 365 days after birth.

### Missing Data

There were no missing data for cannabis or nicotine use, as they were identified using *ICD-9-CM* and *ICD-10-CM* codes. We had a small amount of missing data for covariates, including race and ethnicity (0.04%), prepregnancy BMI (3.7%), insurance status (0.003%), parity (0.1%), and prenatal visits (1.6%). We addressed missingness by performing multiple imputation using chained equations to generate 20 imputed data sets, prior to conducting regression analyses. We pooled estimates according to Rubin’s rule.^23^

### Statistical Analysis

Data were analyzed October 14, 2023, to March 4, 2024. Prevalence of a cannabis-related diagnosis and/or nicotine product use (cannabis, nicotine, and both) was determined. Comparisons of the 4 groups were performed using χ^2^ tests. Multivariable Poisson regression models with robust variance estimators were used to assess the association among maternal cannabis use, nicotine use, or combined cannabis and nicotine use with adverse perinatal outcomes, and adjusted risk ratios (ARRs) were estimated. The regression models evaluated the 3 mutually exclusive exposure categories with reference to controls. Potential confounding variables were chosen based on a priori understanding of variables that have historically been associated with both the covariates of interest and outcomes. Potential confounders identified included maternal age, race and ethnicity, educational attainment, prepregnancy BMI, parity, prenatal visits, health insurance, chronic hypertension, preexisting diabetes, and mental health disorders. We performed sensitivity analyses to check the robustness of our results. First, sensitivity analyses were performed using *ICD-9-CM* and *ICD-10-CM* codes for cannabis-related diagnosis and nicotine (excluding those only identified through nicotine selected on birth records). Although medical cannabis became legal in 2012, recreational cannabis was legalized in 2016. Thus, we performed sensitivity analyses by only using data from 2017 to 2019. Another sensitivity analysis was performed by including patients who reported more than one race. Because of multiple comparisons, statistical significance was set at 2-sided *P* = .005 to decrease type I error rate.^27^ Statistical analyses were performed using Stata, version 17 (StataCorp LLC).

## Results

**Figure 1.**
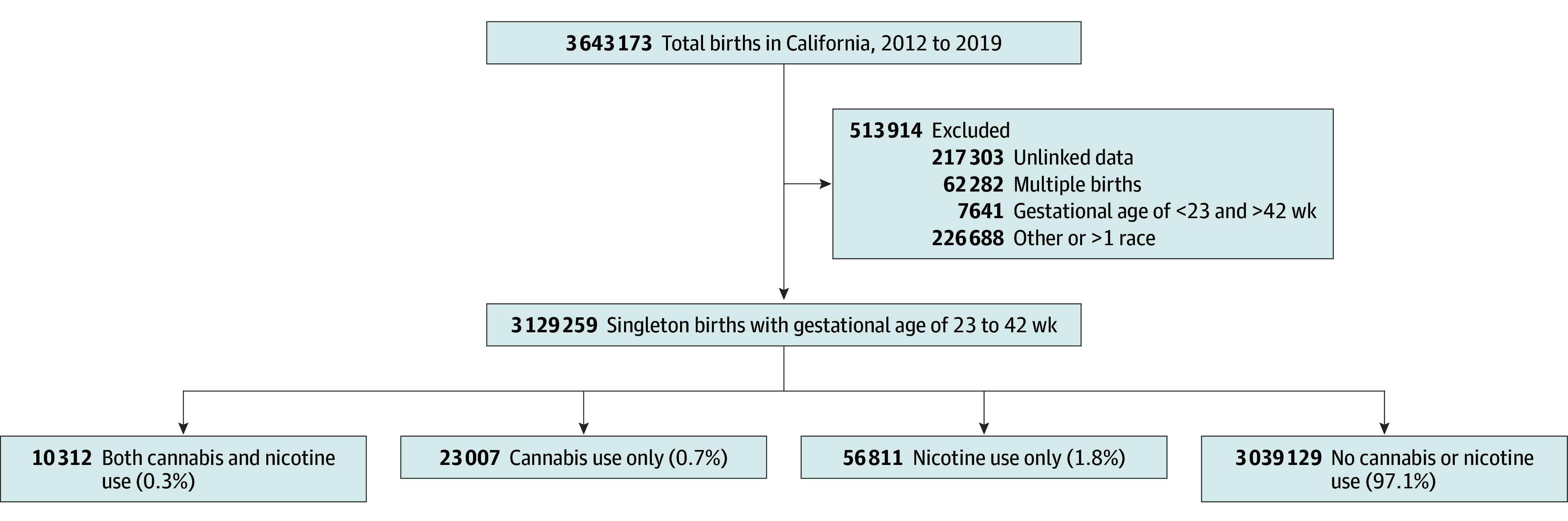
Study Flow Chart

**Figure 2.**
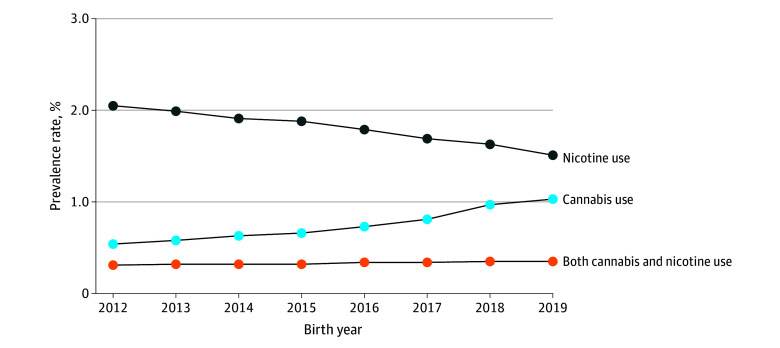
Prevalence Rates of Cannabis and Nicotine Use Among Pregnant Individuals in California

A total of 3 129 259 pregnant individuals met inclusion criteria (mean [SD] maternal age 29.3 [6.0] years) (Figure 1). Among all participants, 0.3% were American Indian or Alaska Native, 13.1% were Asian or Native Hawaiian or Other Pacific Islander, 5.2% were Black, 52.9% were Hispanic, and 28.5% were White. A total of 23 007 participants (0.7%) had a cannabis-related diagnosis, 56 811 (1.8%) used nicotine products, and 10 312 (0.3%) used both cannabis and nicotine. Among 33 319 pregnant individuals with cannabis use, 10 312 (30.9%) also used nicotine. Similarly, nicotine products were used by 67 123 pregnant individuals and of these, 10 312 (15.4%) also had a cannabis-related diagnosis. From 2012 to 2019 in California, the prevalence of cannabis use among pregnant individuals increased (0.5% to 1.0%), nicotine product use decreased (2.1% to 1.5%), and combined use of both cannabis and nicotine remained largely stable (0.3% to 0.4%; *P* < .001 [χ^2^ test]) (Figure 2).

Compared with nonusers, individuals using both cannabis and nicotine were more likely to be White (50.7% vs 27.9%), to have public or no insurance or be self-insured (87.5% vs 52.3%), and to have fewer than 5 prenatal visits during the course of their pregnancy (20.5% vs 2.2%) (Table 1). Patients using both cannabis and nicotine had a higher proportion of mental health disorders, chronic hypertension, and preexisting diabetes compared with controls (Table 1).

**Table 1.** Participant Characteristics^a^

Characteristic	Substance used, % of participants			
	Unexposed (n = 3 039 129)	Cannabis (n = 23 007)	Nicotine products (n = 56 811)	Cannabis and nicotine (n = 10 312)
Race and ethnicity				
American Indian or Alaska Native	0.3	1.2	1.5	1.8
Asian or Hawaiian or Other Pacific Islander	13.4	1.3	3.7	1.2
Black	4.9	22.4	12.4	21.0
Hispanic	53.5	45.8	24.4	25.2
White	27.9	29.2	58.0	50.7
Missing	0.04	0.04	0.1	0.1
Maternal age, y				
<20	5.2	12.5	4.2	7.3
20-34	73.8	79.7	79.3	81.4
≥35	21.0	7.8	16.5	11.3
Educational attainment				
High school or less	41.1	59.9	59.9	65.0
Some college	58.9	40.1	40.1	35.0
Prepregnancy BMI				
Underweight (<18.5)	3.6	5.1	4.9	6.8
Normal weight (18.5-24.9)	44.0	39.8	38.7	42.5
Overweight (25.0-29.9)	25.8	24.2	24.0	22.2
Obesity (≥30.0)	23.1	26.8	27.7	21.9
Missing	3.6	4.1	4.7	6.6
Insurance				
Private	47.7	22.8	19.9	12.5
Public, self-pay, or uninsured	52.3	77.2	80.1	87.5
Missing	0	0	0	0.02
Parity				
Multiparous	60.9	55.0	71.4	65.9
Nulliparous	39.0	44.8	28.4	33.8
Missing	0.04	0.13	0.2	0.3
Prenatal visits				
≥5	96.3	87.9	84.6	75.8
<5	2.2	9.3	12.7	20.5
Missing	1.5	2.7	2.7	3.7
Alcohol use	0.1	2.1	1.3	3.4
Chronic hypertension	1.9	3.9	3.7	4.6
Preexisting type 1 or 2 diabetes	1.3	1.5	1.9	2.3
Mental health disorders	3.5	15.6	12.1	20.6

Abbreviation: BMI, body mass index (calculated as weight in kilograms divided by height in meters squared).

^a^ *P* < .001 for all differences among 4 groups (calculated using χ^2^ test), among data reported as percentages.

### Maternal Outcomes

Rates of hypertensive disease were higher in patients with a cannabis-related diagnosis (12.3%), nicotine use (9.6%), and both (11.2%) compared with controls (7.6%; *P* < .001) (Table 2). Multivariable Poisson regression analyses showed that the risk of hypertensive disease was higher among patients with a cannabis-related diagnosis (ARR, 1.36 [95% CI, 1.32-1.42]), nicotine-product use (ARR, 1.19 [95% CI, 1.16-1.23]), and both (ARR, 1.33 [95% CI, 1.26-1.40]), when compared with controls (Table 3).

**Table 2.** Perinatal Outcomes Among Pregnant Individuals in California, 2012 to 2019^a^

Outcome	Substance used, % of participants			
	Unexposed (n = 3 039 129)	Cannabis-use (n = 23 007)	Nicotine-product use (n = 56 811)	Both (n = 10 312)
Hypertensive disease^b^	7.6	12.3	9.6	11.2
Preterm delivery <37 wk	6.6	12.2	12.0	17.5
Preterm delivery <32 wk	0.8	2.1	1.8	2.9
SMM	1.3	2.3	2.1	2.6
Nontransfusion SMM	0.4	0.8	0.7	1.0
NICU admission	10.0	15.6	17.8	22.5
Small for gestational age	8.5	14.3	13.7	18.0
Respiratory distress syndrome	3.4	5.9	5.5	7.5
Infant death	0.3	0.7	0.7	1.2
Neonatal death	0.2	0.3	0.3	0.6
Postneonatal death	0.1	0.4	0.4	0.6
Hypoglycemia	2.1	3.5	3.0	3.8
Bronchopulmonary dysplasia	0.1	0.2	0.1	0.2

Abbreviations: NICU, neonatal intensive care unit; SMM, severe maternal morbidity.

^a^ *P* < .001 for all differences among 4 groups (calculated using χ^2^ test).

^b^ Includes gestational hypertension and preeclampsia with or without severe features.

**Table 3.** Unadjusted and Adjusted RRs for Adverse Perinatal Outcomes

Outcome	Participant group							
	Unexposed (n = 3 039 129)		Cannabis use (n = 23 007)		Nicotine-product use (n = 56 811)		Combination use (n = 10 312	
	Unadjusted RR (95% CI)	ARR (95% CI)^a^	Unadjusted RR (95% CI)	ARR (95% CI)^a^	Unadjusted RR (95% CI)	ARR (95% CI)^a^	Unadjusted RR (95% CI)	ARR (95% CI)^a^
Maternal								
Hypertensive disease	1 [Reference]	1 [Reference]	1.61 (1.55-1.67)	1.36 (1.32-1.42)	1.26 (1.23-1.29)	1.19 (1.16-1.23)	1.47 (1.39-1.54)	1.33 (1.26-1.40)
Preterm delivery <37 wk	1 [Reference]	1 [Reference]	1.85 (1.79-1.92)	1.47 (1.42-1.53)	1.82 (1.78-1.86)	1.48 (1.45-1.52)	2.66 (1.55-1.78)	1.83 (1.75-1.91)
Very preterm delivery (<32 wk)	1 [Reference]	1 [Reference]	2.59 (2.37-2.83)	1.55 (1.41-1.70)	2.16 (2.03-2.31)	1.36 (1.27-1.45)	3.52 (3.14-3.93)	1.61 (1.43-1.81)
SMM	1 [Reference]	1 [Reference]	1.73 (1.59-1.89)	1.33 (1.22-1.45)	1.62 (1.53-1.72)	1.42 (1.34-1.50)	2.04 (1.81-2.29)	1.46 (1.29-1.64)
Nontransfusion SMM	1 [Reference]	1 [Reference]	1.87 (1.62-2.17)	1.42 (1.23-1.65)	1.72 (1.56-1.91)	1.51 (1.36-1.67)	2.32 (1.91-2.82)	1.63 (1.34-1.99)
Neonatal								
NICU admission	1 [Reference]	1 [Reference]	1.56 (1.52-1.61)	1.33 (1.29-1.37)	1.78 (1.75-1.82)	1.56 (1.53-1.59)	2.25 (2.17-2.34)	1.78 (1.71-1.84)
Small for gestational age	1 [Reference]	1 [Reference]	1.68 (1.63-1.74)	1.46 (1.41-1.51)	1.61 (1.57-1.64)	1.70 (1.66-1.74)	2.11 (2.03-2.20)	1.94 (1.86-2.02)
Respiratory distress syndrome	1 [Reference]	1 [Reference]	1.74 (1.65-1.83)	1.41 (1.34-1.49)	1.64 (1.58-1.69)	1.31 (1.27-1.36)	2.21 (2.07-2.36)	1.56 (1.46-1.67)
Infant deaths	1 [Reference]	1 [Reference]	2.60 (2.23-3.04)	1.65 (1.41-1.93)	2.58 (2.34-2.86)	1.62 (1.45-1.80)	4.46 (3.74-5.33)	2.18 (1.82-2.62)
Neonatal deaths	1 [Reference]	1 [Reference]	2.00 (1.59-2.51)	1.29 (1.03-1.63)	2.09 (1.81-2.42)	1.32 (1.14-1.54)	3.63 (2.82-4.67)	1.76 (1.36-2.28)
Post neonatal deaths	1 [Reference]	1 [Reference]	3.54 (2.86-4.39)	2.16 (1.73-2.70)	3.36 (2.91-3.88)	2.07 (1.77-2.41)	5.79 (4.51-7.44)	2.87 (2.21-3.73)
Hypoglycemia	1 [Reference]	1 [Reference]	1.68 (1.57-1.80)	1.48 (1.38-1.59)	1.45 (1.38-1.53)	1.28 (1.22-1.34)	1.82 (1.65-2.01)	1.49 (1.35-1.65)
Bronchopulmonary dysplasia	1 [Reference]	1 [Reference]	2.81 (2.05-3.86)	1.71 (1.24-2.38)	1.67 (1.28-2.17)	1.06 (0.80-1.41)	2.90 (1.82-4.61)	1.35 (0.83-2.18)

Abbreviations: ARR, adjusted risk ratio; NICU, neonatal intensive care unit; RR, risk ratio; SMM, severe maternal morbidity.

^a^ Adjusted for maternal race and ethnicity, age, education, prepregnancy body mass index, insurance type, parity, prenatal visits, chronic hypertension, preexisting diabetes, and mental health disorders.

Compared with controls, patients with cannabis use, nicotine use, or both had nearly double the rates of preterm delivery at less than 37 weeks (12.2% with a cannabis-related diagnosis, 12.0% with nicotine use, and 17.5% with both compared with 6.6% in controls) (Table 2). Similarly, risk of preterm delivery was higher with a cannabis-related diagnosis (ARR, 1.47 [95% CI, 1.42-1.53]), nicotine use (ARR, 1.48 [95% CI, 1.45-1.52]), and both (ARR, 1.83 [95% CI, 1.75-1.91]) compared with controls. We found similar patterns in the risk of very preterm delivery (<32 weeks) (Table 3).

Higher rates of SMM were also found in patients with a cannabis-related diagnosis (2.3%), nicotine use (2.1%), and both (2.6%) compared with controls (1.3%; *P* < .001) (Table 2). Risk of SMM was higher with a cannabis-related diagnosis (ARR, 1.33 [95% CI, 1.22-1.45]), nicotine use (ARR, 1.42 [95% CI, 1.34-1.50]), and both (ARR, 1.46 [95% CI, 1.29-1.64]) compared with controls. Nontransfusion SMM followed a similar pattern, with the risk highest for combined use of cannabis and nicotine products (ARR, 1.63 [95% CI, 1.34-1.99]) (Table 3).

### Neonatal Outcomes

The rate of infant deaths was 4 times higher in users of cannabis and nicotine combined compared with controls (1.2% vs 0.3%), with rates of 0.7% for those with a cannabis-related diagnosis or use of nicotine products (Table 2). After controlling for confounders, the risk of infant death was 2.18 (95% CI, 1.82-2.62) in individuals with combined use, 1.65 (95% CI, 1.41-1.93) in those with a cannabis-related diagnosis, and 1.62 (95% CI, 1.45-1.80) in nicotine users (Table 3). Although the risk of infant mortality was higher in both cannabis and nicotine users than controls, the risk in individuals who used both was much higher than the risk associated with cannabis or nicotine use alone. Rates of neonatal deaths were 0.3% for those with a cannabis-related diagnosis or use of nicotine products, 0.6% for combined users, and 0.2% for controls (ARR, 1.76 [95% CI, 1.36-2.28]). Postneonatal deaths followed a similar pattern.

Rates of NICU admission were more than double in patients with use of both cannabis and nicotine compared with controls (22.5% vs 10.0%). Risk of NICU admission was highest in those with combined use (ARR, 1.78 [95% CI, 1.71-1.84]) followed by those with nicotine use (ARR, 1.56 [95% CI, 1.53-1.59]) and those with a cannabis-related diagnosis (ARR, 1.33 [95% CI, 1.29-1.37]), compared with controls (Table 3).

Similarly, small for gestational age rates in individuals with combined nicotine and cannabis use were more than 2 times greater than for controls (18.0% vs 8.5%), 14.3% or those with cannabis use alone, and 13.7% for those with nicotine use alone. The risk of small for gestational age was almost 2 times greater in those with combined use (ARR, 1.94 [95% CI, 1.86-2.02]) compared with controls. The risks of respiratory distress syndrome, hypoglycemia, and bronchopulmonary dysplasia followed similar patterns (Table 2).

Sensitivity analyses using only *ICD-9-CM* and *ICD-10-CM* codes showed similar results. Using only *ICD-9-CM* and *ICD-10-CM* codes, a total of 51 590 pregnant individuals used nicotine (reduced from 67 123). Adjusted risk ratios for the adverse outcomes in pregnant individuals with a cannabis-related diagnosis, nicotine use, and cooccurrence were relatively unchanged (eTable 3 in Supplement 1). We further conducted sensitivity analysis by using data from 2017 to 2019 (eTable 4 in Supplement 1) and data including patients of other or more than 1 race (eTable 5 in Supplement 1), and results were unchanged.

## Discussion

In this large cohort study, co-occurrence of a cannabis-related diagnosis and use of nicotine products in pregnancy was associated with a significantly higher risk of several important adverse maternal and neonatal outcomes, including infant death, preterm birth before 37 weeks, infants small for gestational age, and NICU admission, when compared with use of either substance alone. These findings persisted despite controlling for potential confounders such as polysubstance use and demographic factors. Taken together, this suggests a potential adverse synergistic effect on offspring morbidity and mortality from prenatal cannabis and nicotine-product combined exposure compared with prenatal exposure of either substance independently.

Although increased infant death has been consistently reported by studies examining the impact of prenatal exposure to nicotine products or cannabis alone, it has been understudied in the setting of combined use. Maternal nicotine product use in pregnancy is a known risk factor for infant death,^13,28^ and some studies^29,30,31^ have suggested a dose-response association between prenatal nicotine exposure and the risk of infant death. Emerging literature has similarly shown that prenatal cannabis exposure is linked with increased infant mortality. A prior cohort study^32^ noted that the incidence of infant death in the first year of life was greater among pregnancies with maternal cannabis use than those without (1.0% compared with 0.4%; ARR, 1.4 [95% CI, 1.2-1.6]). Another study^33^ also noted that prenatal cannabis use was associated with greater odds of death within 1 year of birth (odds ratio, 1.35 [95% CI, 1.12-1.62]). Consistent with our findings, a previous study^34^ noted an increased risk of stillbirth in pregnancies with positive results of umbilical cord blood testing for THC and maternal serum cotinine (main metabolite of nicotine products). Our results suggest a possible synergistic effect on infant death from combined prenatal use of cannabis and nicotine products and help fill the gap in knowledge.

The association between nicotine product use or cannabis-related diagnosis and preterm birth has also been well-established,^4,5,6,35,36,37^ but the knowledge surrounding a cannabis-related diagnosis with nicotine product use and preterm birth is limited.^38,39^ Our findings are consistent with those of a prior systematic review and meta-analysis^5^ that demonstrated that those who concomitantly use cannabis and nicotine products in pregnancy had an increased risk for preterm delivery when compared with those who did not use either substance (11.4% compared with 5.7%; relative risk, 1.85 [95% CI, 1.21-2.81]). Similarly, 2 prior studies^38,39^ also noted an increased risk of preterm birth rate among pregnancies reporting co-occurring use of cannabis and nicotine products. Although the underlying pathophysiology of preterm birth is not well understood, these aggregate studies suggest a potential effect of co-occurring use of cannabis and nicotine products on preterm birth.

In our study, the prevalence of prenatal cannabis-related diagnoses increased and rates of nicotine product use decreased from 2012 to 2019 in California, similar to the trends reported by other studies within the past 10 years.^14,16,40,41^ Interestingly, despite the decreasing use of nicotine products during pregnancy, a cannabis-related diagnosis and co-occurring use of nicotine products in pregnancy remained stable. Our dataset represents a diverse population of pregnant individuals, but rates of a cannabis-related diagnosis and/or nicotine-product use were lower than the national average of 3% to 8%,^2,42,43^ as well as those of a 2017 Maryland-based study^14^ with 71% non-Hispanic Black pregnant participants that demonstrated 9.0% co-occurring use of cannabis and nicotine products, 12.1% cannabis use only, and 7.8% tobacco use only. This suggests the potential for regional differences in prenatal substance use, especially cannabis and nicotine products, compared with the nonpregnant population.^44^

### Strengths and Limitations

Compared with other studies focused on a cannabis-related diagnosis and nicotine products in pregnancy,^14,16,22^ our study included a sizable and diverse patient population and adjusted for confounders, including substance use and demographic factors. Additionally, our study assessed many clinically relevant maternal, prenatal, and neonatal outcomes that are not consistently reported in the literature. Unlike other large existing studies focused on the impact of cannabis use in pregnancy,^5,45,46^ the data included in our study are derived from the period after legalization of medical cannabis in California.

Our study also has limitations. We relied on *ICD-9-CM* and *ICD-10-CM* codes; thus, the study is subject to misclassification bias and may underestimate the true prevalence of both substances used and their associated outcomes. Additionally, these data come from only 1 state, so the results may not be generalizable to other regions of the US. We were also not able to differentiate the timing, duration, quantity, dose, frequency, or mode of cannabis or nicotine product administration used.

## Conclusions

This cohort study is one of the few, to our knowledge, to examine co-occurring use of cannabis and nicotine products in pregnancy and its association with maternal and neonatal outcomes. We found that individuals who use both cannabis and nicotine products during pregnancy had higher rates of adverse perinatal outcomes, including infants small for gestational age and neonatal and infant death, compared with those who used either substance. These risks remained present after controlling for possible confounders. Although the goal is abstinence from both cannabis and nicotine products in pregnancy, for patients who are unable to achieve this, our study suggests that at least cessation of 1 substance would still be beneficial and may help inform public health policy and clinician counseling. This underscores the urgent need for further studies to characterize the impact of prenatal cannabis and nicotine product coexposure, especially the influence of potency, frequency, and timing, on short- and long-term maternal and offspring outcomes to better educate pregnant individuals on the potential harms of use.

## Notes

Supplement 1. **eTable 1.** Comparison of the Vital Statistics Records Linked to Patient Discharge Data Compare With Those Not Linked **eTable 2.** Source of Variables **eTable 3.** Multivariable Poisson Regression Analyses Using *ICD-9-CM* and *ICD-10-CM* Codes for Cannabis and Nicotine Use (2012-2019) **eTable 4.** Multivariable Poisson Regression Analyses Using California Data (2017-2019) Showing Association of Cannabis and/or Nicotine Use With Adverse Perinatal Outcomes **eTable 5.** Multivariable Poisson Regression Analyses After Inclusion of Patients of Other or More Than 1 Race in California (2012-2019)

Supplement 2.
Data Sharing Statement
