# Supplementary material for: Risk of Adverse Neonatal Outcomes After Combined Prenatal Cannabis and Nicotine Exposure
Source: JAMA Netw Open. 2024 May 7;7(5):e2410151. doi: 10.1001/jamanetworkopen.2024.10151 (PMC11077393; doi:10.1001/jamanetworkopen.2024.10151)
Supplement: Supplement 1. — eTable 1. Comparison of the Vital Statistics Records Linked to Patient Discharge Data Compare With Those Not Linked eTable 2. Source of Variables eTable 3. Multivariable Poisson Regression Analyses Using ICD-9-CM and ICD-10-CM Codes for Cannabis and Nicotine Use (2012-2019) eTable 4. Multivariable Poisson Regression Analyses Using California Data (2017-2019) Showing Association of Cannabis and/or Nicotine Use With Adverse Perinatal Outcomes eTable 5. Multivariable Poisson Regression Analyses After Inclusion of Patients of Other or More Than 1 Race in California (2012-2019) [file jamanetwopen-e2410151-s001.pdf]

Supplementary Online Content

Crosland BA, Garg B, Bandoli GE, et al. Risk of adverse neonatal outcomes after combined prenatal cannabis and nicotine exposure. *JAMA Netw Open*. 2024;7(5):e2410151. doi:10.1001/jamanetworkopen.2024.10151

eTable 1. Comparison of the Vital Statistics Records Linked to Patient Discharge Data Compare With Those Not Linked
eTable 2. Source of Variables
eTable 3. Multivariable Poisson Regression Analyses Using *ICD-9-CM* and *ICD-10-CM* Codes for Cannabis and Nicotine Use (2012-2019)
eTable 4. Multivariable Poisson Regression Analyses Using California Data (2017-2019) Showing Association of Cannabis and/or Nicotine Use With Adverse Perinatal Outcomes
eTable 5. Multivariable Poisson Regression Analyses After Inclusion of Patients of Other or More Than 1 Race in California (2012-2019)

This supplementary material has been provided by the authors to give readers additional information about their work.

© 2024 Crosland BA et al. *JAMA Network Open*.

eTable 1. Comparison of the Vital Statistics Records Linked to Patient Discharge Data Compared With Those Not Linked

	Analytic sample (N=3,425,870)	Individuals not linked (N=217,303)
Race/ethnicity		
American Indian and Alaska Native	0.30%	0.30%
Asian/Pacific Islanders	12.30%	13.00%
Hispanic	49.10%	47.70%
Non-Hispanic white	26.70%	25.40%
Non-Hispanic Black	4.90%	5.10%
Other/Multiracial	6.80%	8.50%
Maternal age (Years)		
<20	5.10%	4.70%
20-34	73.50%	71.70%
≥35	21.50%	23.60%
Education		
High school or less	40.00%	36.30%
Some college	60.00%	63.70%
Pre-pregnancy body mass index (BMI)		
Underweight	3.80%	3.40%
Normal weight	46.10%	45.20%
Overweight	26.50%	27.20%
Obese	23.60%	24.20%
Insurance		
Private	48.20%	57.70%
Public	51.80%	42.30%
Nulliparous	39.00%	39.70%
Prenatal visits (less than five)	2.60%	4.10%
Alcohol use	0.10%	0.00%
Substance related diagnosis	0.90%	0.60%

© 2024 Crosland BA et al. *JAMA Network Open*.

Chronic hypertension	2.10%	1.70%
Pre-existing diabetes	1.30%	1.10%
Mental health disorders	3.90%	1.00%

eTable 2. Source of Variables

Variable	Data source	Notes/ICD-9 codes	ICD-10 codes
Race and ethnicity	Vital statistics		
Age	Vital statistics		
Education	Vital statistics		
Pre-pregnancy body mass index (BMI)	Vital statistics	Calculated from height and pre-pregnancy weight	
Insurance	Vital statistics		
Parity	Vital statistics		
Prenatal visits	Vital statistics		
Alcohol use	Hospital discharge data	291.81, 303.0, 303.9, 305.0, 790.3, 980.x	F10
Chronic hypertension	Hospital discharge data and vital statistics	642.1, 642.2, 642.7, 401.x, 402.x, 403.x, 404.x, 405.x	I10.x, I11.x, I12.x, I13.x, I14.x, I15.x, I16.x, O10.x, O11.x
Pre-existing diabetes	Hospital discharge data and vital statistics	250.x	E10, E11, O24.0, O24.1, O24.3, O24.8
Mental health disorders	Hospital discharge data	300.x, 296.2, 296.3, 311.x, 296.0, 296.4, 296.5, 296.6, 296.80, 296.89	F41, F32, F33, F31
Cannabis-use	Hospital discharge data	305.2, 304.3	F12

© 2024 Crosland BA et al. *JAMA Network Open*.

Nicotine	Hospital discharge data and vital statistics	305.1, 649.0	O99.33, F17
Hypertensive disease	Hospital discharge data and vital statistics	642.0, 642.3, 642.4, 642.5	O13, O14
Preterm delivery<37 weeks	Vital statistics		
Preterm delivery<32 weeks	Vital statistics		
Severe maternal morbidity	Hospital discharge data	Published algorithm using 21 indicators provided by CDC	
NICU admission	Vital statistics		
Small for gestational age	Vital statistics	Published algorithm using gestational age, neonate's gender and birth weight	
Respiratory distress syndrome	Hospital discharge data	769.x	P22
Infant deaths	Vital statistics	Defined as deaths within one year of age	
Neonatal deaths	Vital statistics	Defined as death between 0-28 days of birth	
Post-neonatal deaths	Vital statistics	Defined as death between 28-265 days of birth.	
Hypoglycemia	Hospital discharge data	775.6, 251.0, 251.1, 251.2	P70.3, P70.4, E16.1, E16.2, E15
Bronchopulmonary dysplasia	Hospital discharge data	770.7	P27.1

© 2024 Crosland BA et al. *JAMA Network Open*.

eTable 3. Multivariable Poisson Regression Analyses Using *ICD-9-CM* and *ICD-10-CM* Codes for Cannabis and Nicotine Use (2012-2019)

	Unexposed (n= 3,054,340) aRR (95% CI)[a]	Cannabis use (n= 23,992) aRR (95% CI)[a]	Nicotine use (n= 42,253) aRR (95% CI)[a]	Co-use (n= 9,337) aRR (95% CI)[a]
Maternal outcomes				
Hypertensive disease	Reference	1.36 (1.32, 1.41)	1.28 (1.24, 1.32)	1.34 (1.26, 1.41)
Preterm delivery (<37 weeks)	Reference	1.48 (1.43, 1.53)	1.52 (1.48, 1.56)	1.82 (1.74, 1.90)
Very preterm delivery (<32 weeks)	Reference	1.54 (1.41, 1.68)	1.43 (1.33, 1.54)	1.63 (1.45, 1.84)
Severe maternal morbidity (SMM)	Reference	1.33 (1.22, 1.44)	1.53 (1.44, 1.63)	1.48 (1.31, 1.67)
Non-transfusion SMM	Reference	1.41 (1.22, 1.63)	1.72 (1.55, 1.92)	1.69 (1.38, 2.08)
Neonatal outcomes				
NICU admission	Reference	1.34 (1.30, 1.38)	1.58 (1.55, 1.62)	1.76 (1.69, 1.83)
Small for gestational age	Reference	1.47 (1.42, 1.52)	1.62 (1.58, 1.66)	1.90 (1.82, 1.99)
Respiratory distress syndrome	Reference	1.41 (1.34, 1.48)	1.39 (1.34, 1.44)	1.58 (1.47, 1.69)
Infant deaths	Reference	1.65 (1.42, 1.93)	1.72 (1.53, 1.94)	2.19 (1.81, 2.65)
Neonatal death	Reference	1.37 (1.10, 1.70)	1.44 (1.22, 1.69)	1.67 (1.26, 2.21)
Post-neonatal death	Reference	2.07 (1.69, 2.58)	2.16 (1.82, 2.55)	3.04 (2.33, 3.96)
Hypoglycemia	Reference	1.49 (1.39, 1.59)	1.35 (1.28, 1.42)	1.48 (1.33, 1.64)
Bronchopulmonary dysplasia	Reference	1.69 (1.22, 2.33)	1.24 (0.93, 1.68)	1.42 (0.86, 2.32)

aRR—Adjusted risk ratio, CI-Confidence interval

[a] Adjusted for maternal race and ethnicity, age, education, pre-pregnancy BMI, insurance, parity, prenatal visits, chronic hypertension, pre-existing diabetes, and mental health disorders

© 2024 Crosland BA et al. *JAMA Network Open*.

eTable 4. Multivariable Poisson Regression Analyses Using California Data (2017-2019) Showing Association of Cannabis and/or Nicotine Use With Adverse Perinatal Outcomes

	Unexposed (n= 1,140,467) aRR (95% CI)[a]	Cannabis use (n= 11,194) aRR (95% CI)[a]	Nicotine use (n= 19,364) aRR (95% CI)[a]	Co-use (n= 4,171) aRR (95% CI)[a]
Maternal outcomes				
Hypertensive disease	Reference	1.38 (1.32, 1.45)	1.24 (1.19, 1.29)	1.42 (1.31, 1.54)
Preterm delivery (<37 weeks)	Reference	1.47 (1.40, 1.55)	1.50 (1.44, 1.56)	1.85 (1.72, 1.98)
Very preterm delivery (<32 weeks)	Reference	1.59 (1.39, 1.82)	1.40 (1.25, 1.56)	1.74 (1.45, 2.08)
Severe maternal morbidity (SMM)	Reference	1.29 (1.14, 1.47)	1.51 (1.37, 1.66)	1.55 (1.29, 1.86)
Non-transfusion SMM	Reference	1.46 (1.19, 1.80)	1.58 (1.34, 1.86)	1.80 (1.33, 2.43)
Neonatal outcomes				
NICU admission	Reference	1.35 (1.28, 1.41)	1.71 (1.66, 1.77)	1.82 (1.71, 1.92)
Small for gestational age	Reference	1.47 (1.39, 1.54)	1.63 (1.57, 1.69)	1.90 (1.77, 2.04)
Respiratory distress syndrome	Reference	1.25 (1.17, 1.33)	1.45 (1.38, 1.51)	1.60 (1.47, 1.75)
Infant deaths	Reference	1.82 (1.44, 2.30)	1.20 (0.97, 1.50)	2.32 (1.72, 3.13)
Neonatal death	Reference	1.49 (1.07, 2.09)	1.02 (0.75, 1.38)	1.84 (1.20, 2.83)
Post-neonatal death	Reference	2.27 (1.63, 3.17)	1.46 (1.07, 1.99)	3.05 (2.00, 4.64)
Hypoglycemia	Reference	1.46 (1.33, 1.59)	1.33 (1.23, 1.43)	1.52 (1.33, 1.75)
Bronchopulmonary dysplasia	Reference	1.71 (1.06, 2.78)	1.01 (0.62, 1.63)	1.38 (0.65, 2.97)

aRR—Adjusted risk ratio, CI-Confidence interval
[a] Adjusted for maternal race and ethnicity, age, education, pre-pregnancy BMI, insurance, parity, prenatal visits, chronic hypertension, pre-existing diabetes, and mental health disorders

© 2024 Crosland BA et al. *JAMA Network Open*.

eTable 5. Multivariable Poisson Regression Analyses After Inclusion of Patients of Other or More Than 1 Race in California (2012-2019)

	Unexposed (n=3,257,184) aRR (95% CI)[a]	Cannabis use (n=25,165) aRR (95% CI)[a]	Nicotine use (n=62,201) aRR (95% CI)[a]	Co-use (n=11,397) aRR (95% CI)[a]
Maternal outcomes				
Hypertensive disease	Reference	1.35 (1.31, 1.40)	1.19 (1.16, 1.22)	1.31 (1.24, 1.38)
Preterm delivery (<37 weeks)	Reference	1.36 (1.41, 1.51)	1.47 (1.44, 1.51)	1.81 (1.74, 1.89)
Very preterm delivery (<32 weeks)	Reference	1.57 (1.44, 1.71)	1.33 (1.25, 1.42)	1.61 (1.44, 1.81)
Severe maternal morbidity (SMM)	Reference	1.31 (1.21, 1.43)	1.41 (1.33, 1.49)	1.44 (1.29, 1.62)
Non-transfusion SMM	Reference	1.43 (1.24, 1.64)	1.48 (1.34, 1.63)	1.62 (1.34, 1.95)
Neonatal outcomes				
NICU admission	Reference	1.31 (1.28, 1.35)	1.55 (1.52, 1.57)	1.78 (1.72, 1.84)
Small for gestational age	Reference	1.44 (1.39, 1.48)	1.67 (1.64, 1.71)	1.90 (1.82, 1.98)
Respiratory distress syndrome	Reference	1.41 (1.34, 1.48)	1.31 (1.27, 1.36)	1.60 (1.50, 1.71)
Infant deaths	Reference	1.70 (1.47, 1.98)	1.67 (1.51, 1.84)	2.14 (1.80, 2.56)
Neonatal death	Reference	1.28 (1.03, 1.60)	1.34 (1.16, 1.56)	1.79 (1.41, 2.29)
Post-neonatal death	Reference	2.30 (1.88, 2.83)	2.13 (1.85, 2.46)	2.71 (2.10, 3.49)
Hypoglycemia	Reference	1.46 (1.36, 1.56)	1.27 (1.21, 1.33)	1.47 (1.34, 1.62)
Bronchopulmonary dysplasia	Reference	1.57 (1.13, 2.17)	1.00 (0.76, 1.31)	1.47 (0.95, 2.28)

aRR—Adjusted risk ratio, CI-Confidence interval

[a] Adjusted for maternal race and ethnicity, age, education, pre-pregnancy BMI, insurance, parity, prenatal visits, chronic hypertension, pre-existing diabetes, and mental health disorders

© 2024 Crosland BA et al. *JAMA Network Open*.
