# Supplementary material for: Risk of Adverse Neonatal Outcomes After Combined Prenatal Cannabis and Nicotine Exposure
Source: JAMA Netw Open. 2024 May 7;7(5):e2410151. doi: 10.1001/jamanetworkopen.2024.10151 (PMC11077393; doi:10.1001/jamanetworkopen.2024.10151)
Supplement: Supplement 2. — Data Sharing Statement [file jamanetwopen-e2410151-s002.pdf]

Data Sharing Statement

Crosland. Risk of Adverse Neonatal Outcomes After Combined Prenatal Cannabis and Nicotine Exposure. *JAMA Netw Open*. Published May 07, 2024.
doi:10.1001/jamanetworkopen.2024.10151

Data
Data available: No

Additional Information
Explanation for why data not available: Data will be made available on reasonable request.
